# Swimming Into View: Zebrafish Uncover Targets, Mechanisms, and Therapies for Cadmium Toxicity

**DOI:** 10.1007/s40572-025-00471-0

**Published:** 2025-04-22

**Authors:** Jessica Okutsu, Md Imran Noor, Delia S. Shelton

**Affiliations:** Department of Biology, University of Miami, 1301 Memorial Dr., Cox Science Building Rm 27, Coral Gables, FL 33146 USA

**Keywords:** Cadmium, Zebrafish, Behavior, Molecular mechanisms, Therapies

## Abstract

**Purpose of Review:**

Cadmium (Cd) remains a persistent threat to human and environmental health. To better understand causal relationships between genotype and disease phenotypes, a genetically tractable model, zebrafish (*Danio rerio*) has emerged. We summarize recent empirical evidence on the targets, mechanisms, and potential therapies for Cd toxicity.

**Recent Findings:**

Recent results show that waterborne Cd exhibits organ specific accumulation including in the eye, brain, heart, and gonads triggering oxidative stress, inflammation, gut dysbiosis, and altered methylation patterns that persist across generations. Novel mechanisms of Cd toxicity include the gut-brain axis, ionic antagonism, the Wnt/β-catenin pathway, and epigenetics, leading to potential therapeutics such as probiotics, selenium, and antioxidants.

**Summary:**

Based on the reviewed literature, more studies should examine the effects of dietary Cd on zebrafish behavior, brains, and cardiovascular function. Given that humans and wildlife are chronically exposed to Cd, leading to gonadal Cd accumulation, studies should conduct early-life exposures across the zebrafish lifespan and assess endpoints across generations to capture germline and epigenetic effects and mechanisms. The zebrafish’s biomedical toolkit, along with high-content screening, should be utilized to develop and refine therapies.

**Supplementary Information:**

The online version contains supplementary material available at 10.1007/s40572-025-00471-0.

## Introduction

Cadmium (Cd) is a known carcinogen, mutagen, and immunotoxicant increasing in the environment [1, 2]. Cd is ranked seventh among toxic substances according to the Agency for Toxic Substances and Disease Registry (ATSDR) [3]. Cd is found in nearly everything we eat, drink and breathe. Individuals consuming some rice and vegetables were found to have an average daily Cd intake of 596 µg/d, more than 10 times the provisional tolerated weekly intake (PTWI) [4]. Tissue specific toxicity dominate global standards for chemical toxicity with the PTWI for Cd is based on nephrotoxicity, PTWI of 25 μg/kg/bw/month or 50 μg/d assuming a body weight of 60 kg [4]; thus, other adverse health effects of Cd exposure may be prevalent. Cd’s toxicity is concerning as levels below established thresholds cause adverse effects [4]. Cd accumulates in reproductive tissues, eyes, brain, and heart, and alters methylation patterns (epigenetic mechanism; Table 1) suggesting potential transgenerational effects on vision, behavior and cardiovascular function. Because the structural and functional aspects of neurons and visual systems are conserved across vertebrates [5–7], many parallels can be drawn between humans and non-human animals. Zebrafish have emerged as a valuable model organism for studying Cd-induced toxicity due to their genetic and physiological similarities to humans, rapid development, well-characterized behavioral repertoire in the lab and the field [8–10], ease of genetic manipulation [11], and amenability for testing various concentrations and exposure routes [12–15]. The high degree of genome conservation between zebrafish and humans, along with recent advancements in sequencing technologies, have driven the use of zebrafish to elucidate the causal relationships between genotype and disease phenotypes [16].

**Table 1:** Cadmium concentrations in various tissues and whole bodies of different organisms. Concentrations of Cd detected in various tissues of fish, non-human mammals (mice and rabbits), and humans. The minimum and maximum detected levels of cadmium (measured in micrograms per gram, µg/g) across different studies. Note that the values presented do not represent the absolute highest or lowest possible levels of cadmium in these tissues or the whole body, but rather the detected range within the referenced studies. See supplementary materials for references

	Fish		Mice/Rabbits		Humans	
	Minimum detected (µg/g)	Maximum detected (µg/g)	Minimum detected (µg/g)	Maximum detected (µg/g)	Minimum detected (µg/g)	Maximum detected (µg/g)
Brain	0.03 ^ZF 1^	39.04 ^ZF 2^	~ 0.01* ^M 3^	~ 0.1* ^M 4^	0.003 ^H 5^	0.12 ^H 5^
Eyes	-	-	0.02 ^R 6^	0.04 ^R 6^	0.757* ^H 7^	10.015* ^H 7^
Heart	0.36 ^CG 8^	~ 8 ^LT 9^	0.08063 ^M 10^	59.16301 ^M 10^	0.017 ^H 5^	0.25 ^H 5^
Ovary	~ 2.3 ^ZF 11^	~ 6.5 ^ZF 12^	0.006 ^M 13^	0.025 ^M 13^	0.27^£^ ^H 14^	
Testis	-	-	~ 0 ^M 15^	~ 15 ^M 16^	0.11 ^H 14^	0.565 ^H 14^
Bone	-	-	0 ^M 10^	1.82952 ^M 10^	0.0102 ^H 14^	1.8 ^H 17^
Intestine	~ 2.4 ^ZF 18^	~ 85 ^ZF 12^	0 ^M 10^	14.09108 ^M 10^	0.019 ^H 5^	0.83 ^H 5^
Muscle	~ 0.02 ^ZF 1^	~ 2.0 ^ZF 18^	-	-	0.14 ^H 17^	3.2 ^H 17^
Gill/Lung	~ 0.002 ^ZF 19^	89 ^ZF 12^	1.83 ^M 20^	2.93 ^M 20^	0.003 ^H 5^	1.90 ^H 5^
Liver	~ 0.1 ^ZF 1^	118 ^ZF 12^	0.02153 ^M 10^	49.72359 ^M 10^	0.015 ^H 5^	9.65 ^H 5^
Kidney	~ 0.001 ^ZF 19^	~ 1 ^ZF 19^	0.01 ^M 13^	164.255 ^M 10^	0.62 ^H 5^	61.3 ^H 5^
Larvae/Neonate	0.0133 ^ZF 21^	11.1 ^ZF 21^	-	-	-	-
Eggs	~ 0.001 ^ZF 22^	~ 0.015 ^ZF 22^	-	-	-	-
Adult Whole body	0 ^ZF 23^	~ 11 ^ZF 12^	-	-	-	-

*CG* camouflage grouper; *H* human; *LT* lusitanian toadfish; *M* mouse; *R* rabbit; *ZF* zebrafish; and “–” not available

*Concentrations were reported in different parts of the organ, so the values reflect their sums. £ Median reported

Here, we review recent findings on the effects of Cd on the zebrafish visual, cardiovascular, and nervous systems. We synthesize studies to identify major themes, knowledge gaps, and promising research avenues. We will explore specific topics including the power of the zebrafish model for toxicology studies, variation in Cd accumulation across tissues and different routes of exposure, adverse visual effects, cardiovascular toxicity, neurotoxic impacts, and other interactions like the gut-brain axis. We next discuss mechanisms of Cd toxicity including inflammation, common signaling pathways affected (Wnt, metallothionein), oxidative stress, and epigenetics. We end by highlighting how zebrafish can aid in developing novel therapies.

## Cd Accumulation and Distribution in Zebrafish

**Fig. 1:**
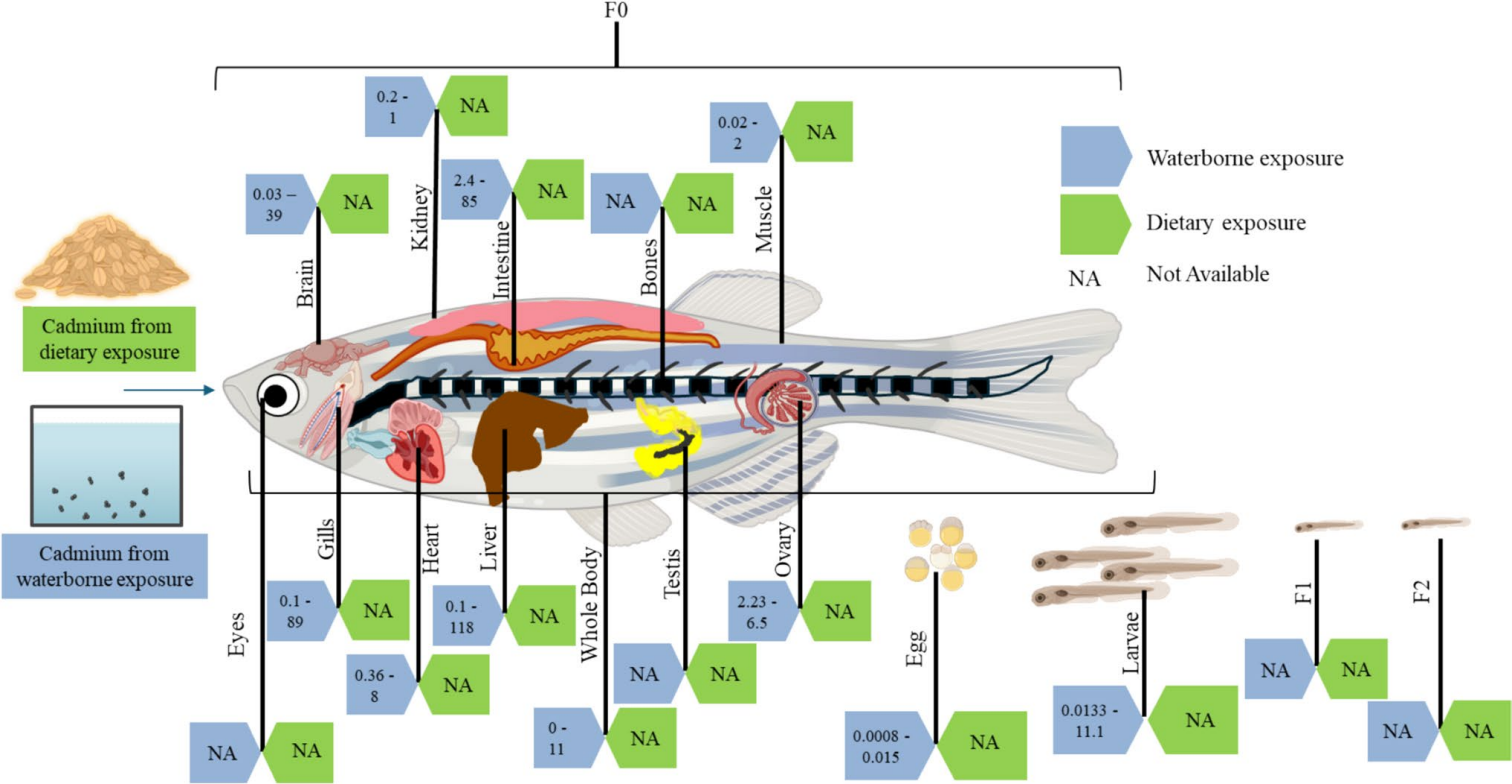
Cadmium bioaccumulation in different organs in zebrafish: This diagram provides a detailed visualization of Cd bioaccumulation in various zebrafish organs. We distinguish the Cd bioaccumulation between dietary (denoted in green boxes) and waterborne (denoted in blue boxes) exposure routes. The values in each box indicate the minimum and maximum levels detected in μg/g in various studies (see Table 1 and Supplementary Materials for references). NA indicates that the bioaccumulation data is not available Created with Biorender

The bioaccumulation of Cd in living organisms presents a significant scientific challenge, as identifying and predicting the accumulation patterns remain highly complex. Research on Cd accumulation has traditionally concentrated on waterborne exposure, whereas studies addressing dietary exposure are notably less common (Fig. 1). Studying chronic dietary exposures is critical for human health because diet is the primary source of Cd accumulation in humans [4, 17].

Zebrafish are ideal for investigating the toxicodynamics of Cd because they are amenable to studying the two main Cd exposure routes: water and diet, and the tertiary route, air [12, 18, 19]. Most studies on Cd toxicity in zebrafish and other model systems have focused on short to sub-chronic waterborne exposures affecting the gills, liver, kidney, and whole body [19–22]. Cd tends to accumulate over time in an organ-specific manner with rapid accumulation in the gills (89 μg/g in 12 h), and slower but higher build up in the liver (89 to 118 µg/g in 72 h) [20]. Acknowledging the extensive research on Cd bioaccumulation in the liver, kidneys, intestine, and gills from waterborne exposure, we will focus on the less studied organs and exposure routes.

Previous studies have reported that Cd is transported in the blood and damage in various organs, including the eyes, brain, heart, bones, and gonads, across different organisms, including humans [14, 23–28]; however, a limited number of studies investigate the distribution and bioaccumulation of Cd in zebrafish organs (see Table 1). For example, recent work reported ~ 11 and ~ 15 µg/g Cd accumulation in the brain and ~ 5.8 and ~ 6.5 µg/g Cd in the ovaries of zebrafish exposed to < 0.5 mg/L and 1.0 mg/L waterborne Cd with accumulation increasing with exposure duration and concentration [20, 29]. Gonadal Cd accumulation may lead to alterations in epigenetic modifications, such as changes in DNA methylation or histone modifications, potentially transmitting to future generations. However, there were a paucity of studies documenting Cd accumulation in subsequent generations (germline – F1 or epigenetic exposure – F2 or further) [30]. Additional studies on Cd accumulation in the male zebrafish reproductive tract are needed to enhance the understanding of reproductive toxicity of Cd. Identifying sex-dependent patterns of Cd accumulation and toxicity in zebrafish will allow us to determine if zebrafish can be used as a model for human sex-dependent Cd accumulation and further undercover underlying mechanisms. It is crucial to identify the patterns of Cd accumulation and toxicity in zebrafish from various sources and routes because this would help explaining the toxicological profile and anchor phenotypic endpoints to bioaccumulation.

## Cd Induces Visual Deficits

Patients with Age-related Macular Degeneration (AMD) visual pathologies who also smoke tobacco have more Cd accumulation in the eye than controls with near ubiquitous presence of Cd in the retinal pigment epithelium and choriocapillaris with detectable accumulation of Cd in the retina using Inductively Coupled Plasma-Mass Spectrometry (ICP-MS) [31], and in the neural retina and optic nerve head using Laser Ablation-Inductively Coupled Plasma-Mass Spectrometry (LA-ICP-MS) [32]. In addition, a substantial portion of the non-smoking and non-AMD afflicted population also exhibit Cd in their eyes [31]. Cd accumulation in the eye has also been confirmed in *in vivo* models such as rats and zebrafish (Table 1) [33]. Cd localization in the eye depends on its form: ionic Cd is localized in the eye, whereas Cd selenide quantum dots have a more uniform distribution on the surface of larval zebrafish [33].

Zebrafish’s color vision and visual reliance offer advantages for studying visual dysfunction over mammalian models like mice and rats [6]. A battery of translatable behavioral paradigms can allow for the identification of Cd effects on the visual, motor, and central nervous systems [6, 34]. Adult and larval zebrafish exposed to less than 1 µg/L of Cd show visual deficits in optomotor and photomotor tasks [25, 35] with cascading effects on their behavioral repertoire including social networks, boldness, and cohesion [13, 25]. Deviant performance in a photomotor assay is attributed to malfunction in the photoreceptors or ganglion cell layers that are vital for photoelectric transmission [34]. Histological assessments reveal that Cd has demonstrable degenerative effects on the choroid, retinal pigment epithelium and photoreceptor layers of the retina [36]. Cd may affect eye morphology by upregulating the melatonin gene *mtnr1c* (corresponding to melatonin receptor 3), which helps regulate fluid pressure in the eye [37]. The impact of Cd on retina without accumulation has not been convincingly demonstrated, because most studies do not concurrently assess Cd accumulation and visually-guided behavior. Cd may alter the blood-retina barrier in order to penetrate the eye [38] and cause cell loss in all retina cell types in zebrafish [36].

Cd’s effect on the visual system has led to disruptions in behavioral rhythms. A typical paradigm to assess visually-guided behavioral rhythms includes presenting zebrafish with 1–5 min intervals of light and dark cycles and then assessing the distance moved [34, 35]. Control fish show greater activity in the light cycle than in the dark cycle, whereas Cd-exposed zebrafish show similar levels of activity in the light and dark cycles [35, 37]. Cd disrupts light–dark cycle responses, possibly by affecting clock1b, clock2 and cry1b genes [37]. Cd exposure leads to arrhythmic expression of clock2, which combines with *bma1* to initiate a transcription and translation feedback loop, subsequently downregulated by *cry1b* (repressor) as part of the 24 h cycle in zebrafish [37]. Many visually-guided behavioral assessments of Cd toxicity require locomotion, necessitating a distinction between visual and motility effects. Motility issues may stem from lethargy due to compromised glycogen reserves or skeletal and cardiac muscle defects [36, 39].

## Cd Compromises the Cardiovascular System

Cardiovascular disease is a critical public health issue causing an estimated 18 million deaths globally each year [40, 41]. Recently, the American Heart Association declared that pollution exposure is an overlooked risk factor for cardiovascular disease [42, 43]. To reduce the loss of life due to cardiovascular disease, fastidious study of the effects of Cd on heart function in a model amenable to toxicology assessments is needed. Zebrafish have an 85% success rate and an excellence designation for identifying human-relevant cardiovascular toxins [44]. Due to the optical clarity of the zebrafish, they are amenable to manual and automated high content machine learning assessments of heart rate, stroke volume, ejection fraction, and cardiac function. Cd’s effects on the zebrafish cardiovascular system are concentration and life-stage specific [45]. Common outcomes of Cd on the zebrafish cardiovascular system include edema and alterations in cardiac function. Though studies show mixed effects on heart rate with some reporting an increase [45], decrease [46], or no effect on heart rate for Cd only exposures [46]. Cd accumulates in the blood of fish, leading to its perfusion into the heart and other organs (Table 1). More generally, changes in cardiovascular function may result from Cd interference with Na^+^/K^+^ ATPase, myosin heavy chain, L-type Ca^2+^ channels, and zinc homeostasis, and activation of the oxidative stress pathways [47].

An area ripe for exploration is understanding Cd’s effect on cardiac remodeling. Zebrafish have the astute ability to regenerate their heart. This ability contrasts starkly with mammalian models who lack cardiac regeneration ability in adulthood with varying degrees of regenerative abilities in neonates [48]. A benchmark study showed that zebrafish can fully regenerate their hearts within 60 days of a 20% ventricular amputation [49]. However, zebrafish with mutations in the Mps1 mitotic checkpoint, a cell cycle regulator, fail to regenerate their hearts and form scars instead [50]. Pairing physiological endpoints with assessments of mRNA expression of cardiac-development and cardiac remodeling related genes would enhance our mechanistic understanding of Cd cardiotoxicity, aiding therapy development and providing a foundation for translational studies [51, 52].

## Adverse Effects of Cd on the Zebrafish Brain: Neural Pathway, Structure, Neurodevelopment, and Gut Microbiota Changes

**Fig. 2:**
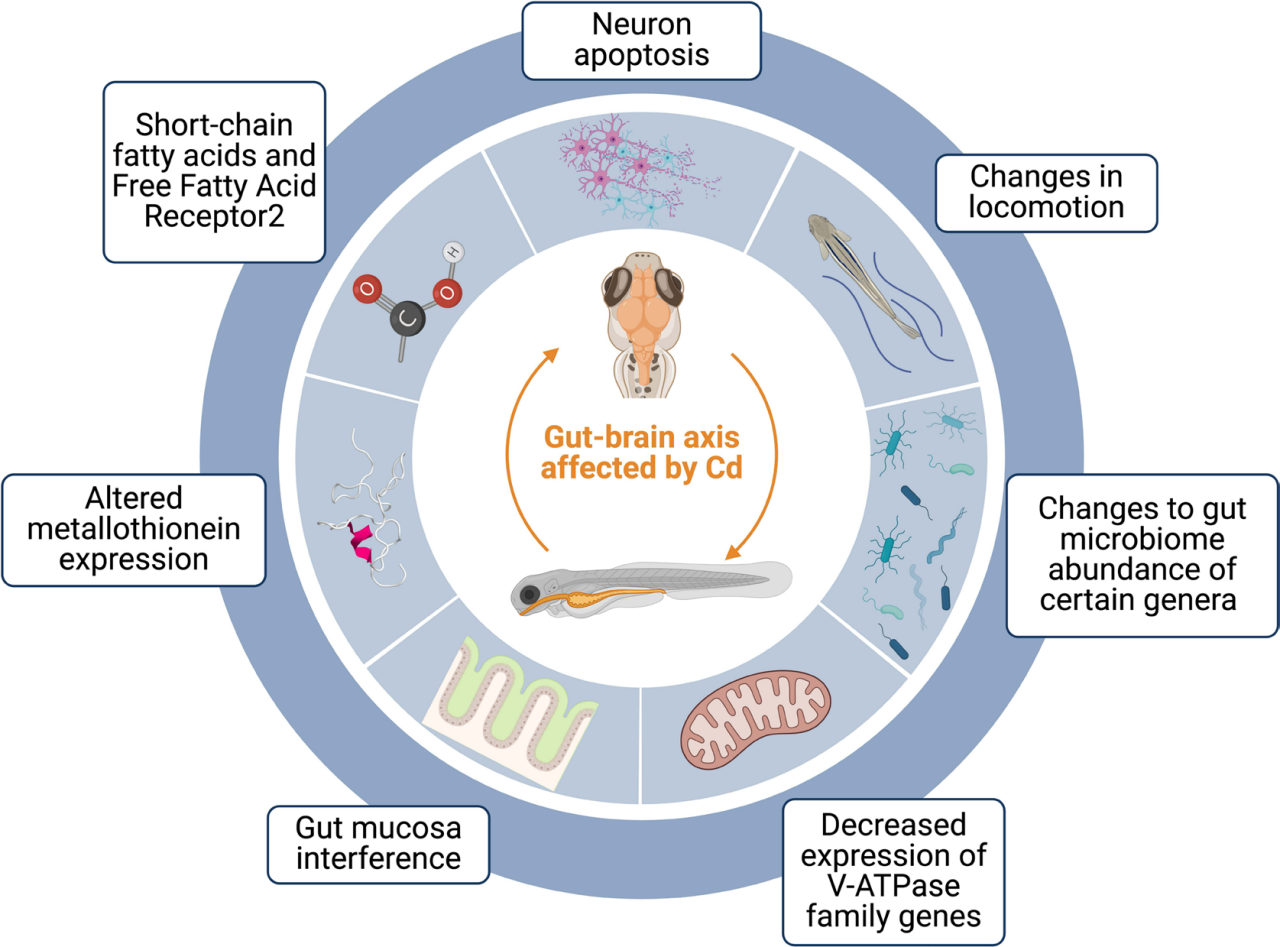
Cd has recently been shown to affect multiple areas involved in the gut-brain axis. Created with Biorender

Cd affects bodily function through disrupting several pathways that lead to inflammation, oxidative stress, apoptosis, organ toxicity, and ultimately organ failure. Describing the mechanisms of Cd toxicity in the brain can provide a clearer connection to its behavioral consequences and enhance the understanding of Cd's impact on other targeted organ systems. Here, we discuss new developments in well-studied mechanisms (e.g., Wnt/β-catenin signaling pathway) and novel mechanisms (e.g., gut dysbiosis) for Cd toxicity in the brain with consequences for behavior (Fig. 2).

Cd exposure disrupts various neural mechanisms in zebrafish, particularly the Wnt/β-catenin signaling pathway, contributing to neuroinflammation, and altered neural function [53, 54]. Cd exposure affects gene expression (Table 2). The Wnt/β-catenin pathway plays a crucial role in mitigating Cd-induced neurotoxicity. The use of TWS119, a glycogen synthase kinase 3 beta (GSK3β) inhibitor and Wnt pathway activator, upregulates β-catenin expression and activates the Wnt/β-catenin pathway, alleviating Cd’s adverse effects in zebrafish embryos [53, 55]. Across multiple studies, disruption of the Wnt/β-catenin pathway emerges as a central mechanism underlying the Cd’s neurotoxic effects, suggesting its potential as a therapeutic target [53–55]. Cd also affects other signaling cascades, such as the Notch pathway [55] and the Hedgehog pathway [54], highlighting the complex network of molecular interactions involved in Cd neurotoxicity.

Cd exposure induces structural changes in the zebrafish brain, especially in the telencephalon and cerebellum, affecting neuronal connections, synaptic remodeling, neuroinflammation, leading to cerebral hemorrhaging, increased blood–brain barrier permeability, and abnormal vascular formation likely due to elevated production of vascular endothelial growth factor [53]. *In vivo* and in vitro experiments reveal altered cell–cell junctional morphology likely through disrupting the proper localization of VE-cadherin and ZO-1, key components of adherens and tight junctions, respectively, following Cd exposure. The inhibition of PTPase activity mediated by Cd-induced ROS is suggested to underlie these deficits. Pretreatment with a ROS production inhibitor, diphenylene iodonium (DPI), partially recovered the Cd-induced inhibition of PTPase activity and alleviated cerebral hemorrhage in zebrafish, confirming the role of ROS in Cd-induced blood–brain barrier dysfunction [56].

Cd exposure during embryonic and larval stages of zebrafish development has long-term consequences for brain development [57]. Exposure to Cd leads to structural and functional abnormalities, delayed early embryonic development, reduced structural complexity of trigeminal ganglion neurons, and downregulation of the expression of genes related to neurodevelopment and differentiation [53]. Cd exposure affects calcium homeostasis within neurons by disrupting the function of voltage-gated calcium channels and calcium-binding proteins, leading to altered dopamine signaling; furthermore, Cd contributes to neurotoxic effects by influencing dopamine neurotransmitter release and synaptic transmission [58]. Cd also alters serotonin, noradrenaline hydrochloride, and glutamate neurotransmitter systems, leading to changes in synthesis, release, and signaling, which are linked to behavioral consequences such as altered swimming patterns, and abnormal locomotion [14, 39, 59].

Disruption of the gut microbiome by Cd exposure has been shown to exacerbate its neurotoxic effects (Fig. 2) [60]. Cd exposure alters the gut microbiota in zebrafish, leading to an increase in isobutyric acid and changes in short-chain fatty acids and FFAR2 expression, which is involved in resolving inflammation as part of the gut-brain axis [60, 61]. In addition, goblet cells, which synthesize mucus in the gastrointestinal tract, show reduced Alcian blue staining, indicating altered mucin composition, and increased N-acetyl-glucosamine in their cytoplasm after exposure to 25 μM and 100 μM Cd compared to controls, with the effects being more pronounced in the anterior intestine than the mid intestine [62]. Cd exposure at 5 μg/L for 7 days significantly changes the relative abundance of *Phascolarctobacterium, Candidatus Saccharimonas*, and *Blautia* in the gut microbiome of adult zebrafish compared to controls [14]. The gut-brain axis is emerging as a key player in Cd neurotoxicity, with studies consistently demonstrating alterations in gut microbiota composition, mucin production, inflammatory signaling, and decreased locomotor activity [14, 60–62]. However, the precise mechanisms linking gut dysbiosis to neurological impairments require further investigation. The extent to which these molecular and symbiotic mechanisms underlie other types of organ toxicity should be studied to identify areas of convergence and divergence in cadmium triggered organ toxicity [63].

## Cd Exposure and Oxidative Stress in Zebrafish

**Fig. 3:**
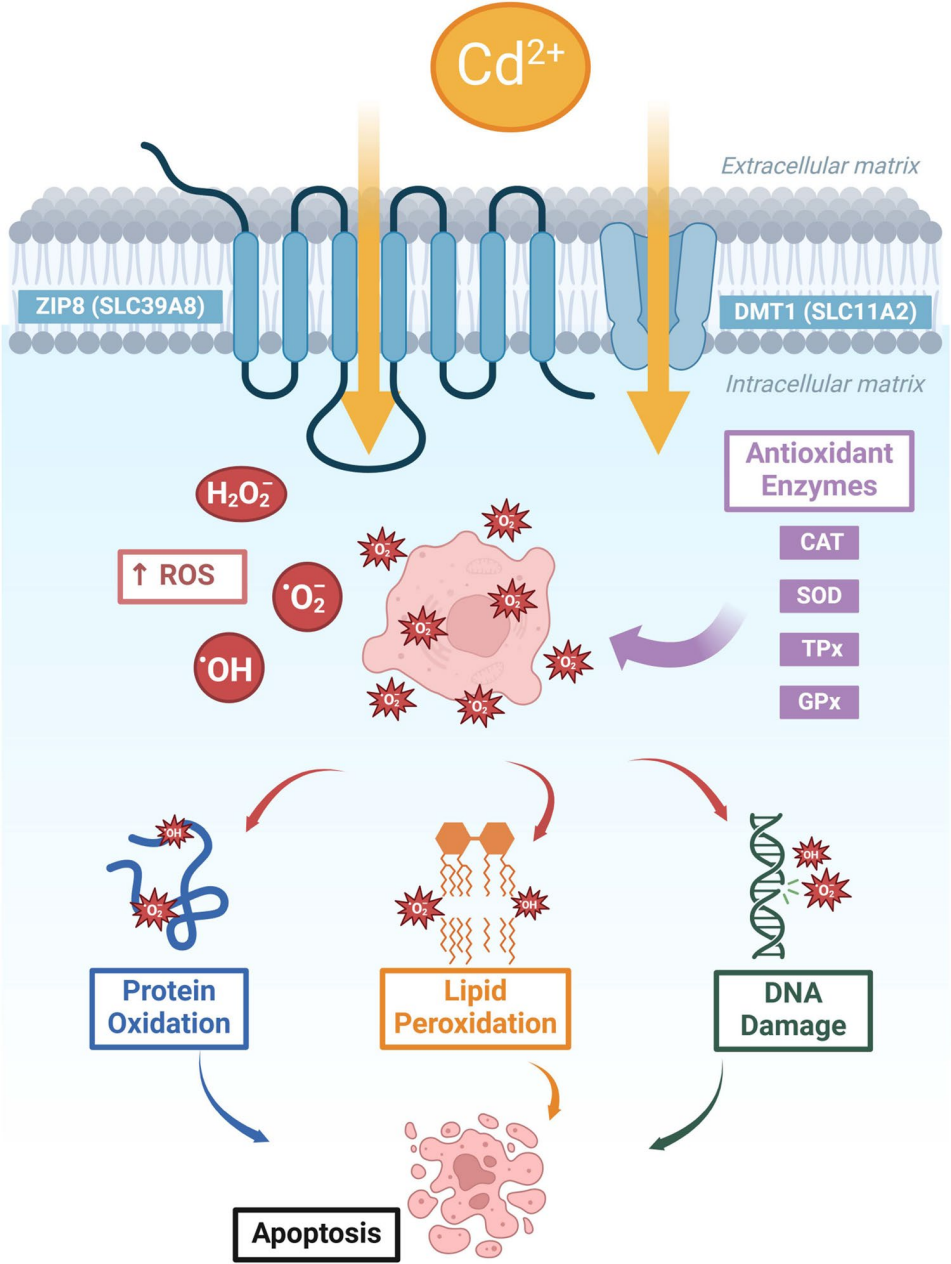
Mechanism of cadmium-induced oxidative stress in cells. An illustration of the biochemical pathways through which cadmium induces oxidative stress in cells, with a focus on the production of reactive oxygen species (ROS). Cadmium enters the cell primarily through transporters such as DMT1 (SLC11A2) and ZIP8 (SLC39A8), Upon entry, Cd facilitates the generation of ROS, including superoxide (O₂⁻) and hydroxyl radicals (OH), which are key players in oxidative stress. These ROS interact with cellular components, resulting in protein oxidation, lipid peroxidation, and DNA damage, culminating in cell death through apoptosis. Antioxidant enzymes such as Catalase (CAT), Superoxide Dismutase (SOD), and Thioredoxin and Glutathione Peroxidases TPx, GPx play critical roles in mitigating the effects of ROS [68]

Cd exposure is linked to reduced levels of necessary antioxidant enzymes, such as catalase, lowered glutathione and disruption of zinc homeostasis, which contribute to developmental anomalies and reproductive difficulties [2, 24, 64–66]. Cd exposure also increases lipid peroxidation (a marker of oxidative damage) in the liver and brain, indicating a defensive cellular response against free radical-induced toxicity in zebrafish [64, 67]. These biochemical changes highlight Cd's detrimental effects on the zebrafish's oxidative status. Oxidative stress is a condition characterized by an imbalance between the production of reactive oxygen species (ROS) and antioxidant defenses. The proposed mechanism (Fig. 3) is that physical stress gives rise to free radicals, which scavenge electrons from cellular molecules, creating a redox state inside the cell [68–70]. Zebrafish exposed to Cd (1.0 mg/L for 16 days) exhibit downregulation of Nrf2 mRNA expression when co-exposed with mercury, but not for independent Cd exposures (see Table 2). This protein plays a vital role in protecting cells from oxidative damage [71]. Metallothioneins play a crucial role in detoxifying Cd by binding to the metal ions and limiting their harmful effects on cells. The expression of metallothionein may modulate cellular responses to Cd, influencing the degree of damage and oxidative stress [72]. Cd exposure upregulates the expression of metallothionein-related gene families in a dose-dependent manner, as demonstrated in human embryonic stem cell-derived cerebral organoids [73]. Thus, there are several molecular pathways involved in antioxidant defense.

**Table 2:** Differential gene expression due to Cd-induced changes. Summary of the differential expression of various genes in response to Cd exposure, including the associated, functions, pathways, tested concentrations, exposure durations, routes of exposure, and model organisms used in the studies. See supplementary materials for references

Downregulated genes	Upregulated genes	No effect	Pathway/ Function	Cd concentrations	Exposure duration	Route of exposure	Model organism	Author
*atp6v1g1, atp6v1b2, atp6v0cb*	*atp6v0a1b*		V-ATPase family genes	0, 0.05 μmol/L (0.005 mg/L), 0.1 μmol/L (0.01 mg/L), and 0.2 μmol/L (0.02 mg/L)	Up to 5 days	Water	Zebrafish	[24]
*FFAR2*			Free fatty acid receptor 2	0, 5 μg/L	7 days	Water	Zebrafish	[25]
*mao, slc6a4a*	*bdnf, tph1b, tph2*		Serotonergic system and social behavior	0, 1.25, 2.5 and 5 μg/L	7 days	Water	Zebrafish (larval)	[26]
axin2, ccnd, lef1, myca, sp52,, wnt10b	*wnt10b*	*β-cateni, gsk3β, wnt4a*	Wnt/β-catenin pathway	0 μg/L, 100, μg/L	6 days	Water	Zebrafish (larval)	[27]
*axin2, β-catenin, ccnd, lef1, myca, sp52, wnt4a, wnt10b*	*gsk3β*		*Wnt/β-catenin pathway*	0 μg/L, 200 μg/L	6 days	Water	Zebrafish (larval)	[27]
*CCDC39, CCDC11 4, CFAP74, DNALI1, RSPH1*	*CALCA,HMOX1, MT1M, MTIF, MT1H, MT1E, MT1G,MT1B,MT2 A, MT1A, SLC7A11, SLC30A1, SLC30A2, TAC1*		Neurodevelopment, ciliogenesis, metal detoxification	0, 40, 80 μM	24 h treatment applied on day 41	Water	Human cerebral organoids	[28]
*cat, GPx1a, Mn-Sod*	*Caspase-3, Caspase-9, C-jun*		Antioxidant pro-apoptosis and pro-inflammatory	2 μg/L	4 to 144 hpf	Water	Zebrafish (embryo)	[29]
		*CAT, NrF2, SOD*	Antioxidant response	1 mg/L	16 days	Water	Zebrafish	[30]
*ErbB-4*	*Dnmt1, Dnmt3, ErbB-3*		ErbB signaling, de-novo methylation	0, 0.0089, 0.089 μM	Up to 24 hpf	Water	Zebrafish (larval)	[31]
*Dnmt3, ErbB-4*	*Dnmt1, ErbB-3*		ErbB signaling, de-novo methylation	0, 0.89 μM	Up to 24 hpf	Water	Zebrafish (larval)	[31]
*Cu/Zn-SOD, cat, IL-6, CTR1*	*IL-1β, INOS*	*HSP70, ZnT8*	ATP binding, protein folding, ferroptosis	0, 197 μg/L	7 days	Water	Zebrafish	[32]
*atp7a, atp7b, cat, hsp70, Sod1, znt5,zip7*	*IL-6, mt2, P65*		Stress response, immune response, metal transport	100, 200 μg/L	4 days	Water	Zebrafish	[33]
*CYP1A, CY1B1, CY1C1, CY1D1, CYP1C2, CYP3A65 CY1B1*			Xenobiotic metabolism	5, 10 μg/L	3-144 hpf	Water	Zebrafish (larval)	[34]
		* CYP1A, CYP1C1, CYP1C2 CYP1D1, CYP3A65*	Xenobiotic metabolism	0, 200, 400 μg/L	24 h	Water	Zebrafish	[34]
*alpl, bmp2b, col10a1, oc1, sparc, spp1 Bcl-2, P53*			Bone development	1 μg/L	20 days	Water	Zebrafish (juvenile)	[35]
			Tumor suppression, apoptosis	5, 15, 30 μM	3, 6, 12, 24 h	Water	Zebrafish (cells)	[36]
*rad51*			DNA repair	5, 15, 30 μM	3, 6, 12, 24 h	Water	Zebrafish (cells)	[36]
*Bcl-2*	*atf4, bax, cas-3,cas-8, cas-9, cat, chop, gpx, hspa5, hsp70, mt2, P53, perk, sod1, xbp1*		Apoptosis, ER stress	5, 10, 20 μg/L	48 day	Water	Zebrafish	[37]

## Cd Exposure and Genotoxicity to Zebrafish

A genotoxin is a substance that can damage the genetic material within cells. The outcomes of genotoxicity can vary, from minor DNA abnormalities to extensive damage that compromises the integrity of the genome and can result in significant health issues [74–76]. The International Agency for Research on Cancer and others have affirmed that ionic Cd induces genotoxic effects across various types of eukaryotic cells, including those of zebrafish and humans [1, 77]. Exposure to Cd may lead to various types of DNA damage in zebrafish, including point mutations, large rearrangements, and structural alterations Table 2 [77]. Reported effects include increased cell death, cell cycle alterations (manifested as an increased cell population in the sub-G1 phase and a decrease for G2/M with no changes in S phase in Cd treatments), which can lead to decreased cell proliferation [78, 79]. While the genotoxicity of Cd in zebrafish is well-established, its effects extend beyond DNA damage to include epigenetic alterations that control gene expression without changing the DNA sequence [80, 81]. Together, these studies show that Cd leads to genotoxicity through DNA damage [77] and epigenetic alterations.

## Cd Exposure and Epigenetic Alteration to Zebrafish

Cd exposure, along with other metals, can directly alter the epigenetic state of the genome. Epigenetic changes involve heritable modifications in gene expression without altering the DNA sequence. Common epigenetic alteration mechanisms include reversible DNA methylation patterns and histone modifications which regulate gene expression. Aberrations in these epigenetic patterns can lead to various clinical outcomes, including cancer, genetic disorders, autoimmune diseases, renal disease, altered neurobehavior, and aging in humans, zebrafish and other species [82–86]. DNA methylation, an essential process for developing embryos, is one of the epigenetic mechanisms affected by Cd toxicity [87]. Zebrafish embryos exposed to 0.089 µM of Cd display expression of DNA Methyltransferases (DNMTs) as *DNMT1* was upregulated, whereas *DNMT3* was downregulated [87]. Cd exposure altered DNA methylation in genes related to ErbB, calcium, MAPK, PI3K/AKT/mTOR, and VEGF pathways, impacting proliferation, differentiation, apoptosis, and transcriptional regulation [87, 88].

**Fig. 4:**
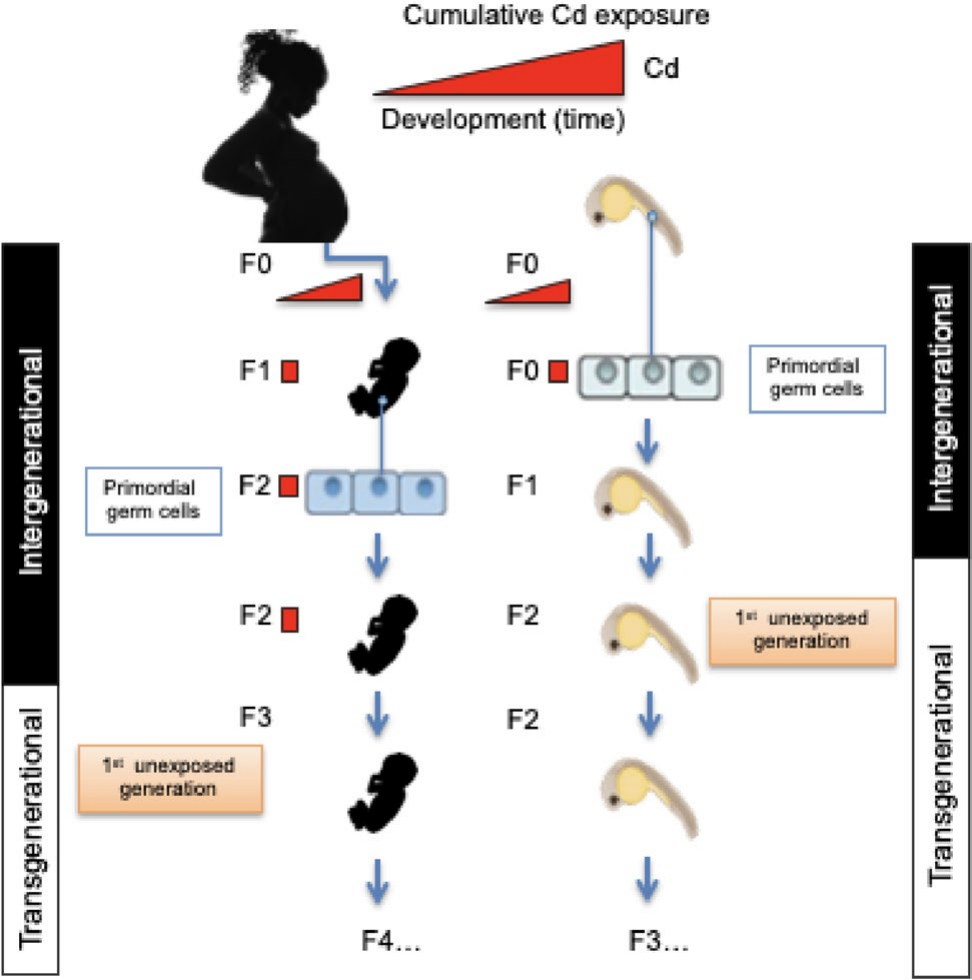
Exposure paradigm for evaluating transgenerational effects of Cd in zebrafish with a comparison to a human exposure scenario. The Cd indicated in red is accumulated across the lifetime. For each generation directly exposed to Cd there is a red box. The diagram shows that intergenerational (germline) effects span F0-F2 generations for humans, and F0-F1 generations for zebrafish, whereas transgenerational (or epigenetic) effects can begin to be observed in the F3 generation for humans and the F2 generation for zebrafish

As with most multi-generational studies, inter-generational studies of Cd toxicity dominate with fewer transgenerational studies of Cd-toxicity [30]. Zebrafish can help bridge this gap because they require only two generations to achieve a non-germline exposure to chemicals, whereas mammalian models require three generations (Fig. 4). This is because zebrafish eggs are externally fertilized, which limits context-dependent epigenetic regulation to the F1 generation, while the F2 generation of either sex is considered transgenerational or affected by germ-line dependent epigenetic regulation. In a series of articles, Pierron and colleagues, report on epigenetic, mortality, and sex-dependent effects of larval zebrafish exposed to Cd in the F0 and F3, but not for other endpoints in subsequent generations (F1-F3) [80, 81, 89]. This exposure paradigm led to transgenerational disorders in *esr1* and *vitellogenins* transcription, abnormal growth, mutation in exon 3, an exon implicated in obesity in mammals, and feminization of offspring. DNA methylation changes were associated with genetic (e.g., CpG-SNPs) and phenotypic changes [81]. For example, * fox12a/dmrt1* methylation levels were influenced by Cd and housing density, leading to skewed sex ratios up to the F3 generation [89]. Further research is needed to elucidate the full range of effects these genetic alterations may have on subsequent generations. Understanding the long-term impacts of Cd exposure will help in assessing the potential risks and developing mitigation strategies. This will also provide deeper insights into how environmental contaminants influence genetic and phenotypic variations across generations.

## Mitigation and Future Directions

Zebrafish models have been used to investigate the therapeutic effects and mechanisms of action of various drugs and natural compounds against bacterial meningitis, pulmonary inflammation, atherosclerosis, and inflammatory bowel disease. Several therapies have been tested in various organisms to mitigate Cd toxicity across multiple organ systems (Table 3). These include investigating potential protective agents or interventions, such as chelation therapy, dietary supplements, antioxidants, and gene therapy [60, 90, 91]. Here, we discuss some of the treatments and their mechanisms on specific target organs.

**Table 3:** Therapies tested in various organisms for cadmium toxicity. See supplementary materials for references

Therapy	Target	Model organism	Clinical trial (Y/N)	Form of administration	Author
*Tamarindus indica* (TM) Coenzyme Q10	Hepatorenal damage	Rat	N	Diet	[38]
*atp6v0cb* overexpression	Neurotoxicity (i.e., locomotion and apoptosis of neurons)	Zebrafish	N	RNA injection	[24]
TWS119	Cell cycle arrest and apoptosis, behavior, activity, and neurodevelopment	Zebrafish	N	Water	[27]
Zinc	Hepatotoxicity	Zebrafish	N	Water	[39]
Zinc	Oxidative stress, inflammation in liver	Zebrafish	N	Water	[33]
Zinc	Neurotoxicity (i.e., cholinergic neurons, oxidative stress, apoptosis, neurite outgrowth)	SH-SY5Y cells	N	Water	[40]
Selenium	Neurotoxicity (i.e., cholinergic neurons, oxidative stress, apoptosis, neurite outgrowth)	SH-SY5Y cells	N	Water	[40]
Selenium	Cardiotoxicity, heart rate, pericardial edema	Zebrafish	N	Water	[41]
AL-TPSPH (Tree peony seed protein hydrolysate by Alcalase)	Oxidative damage, inflammation, apoptosis, hatching, morphology, survival	Zebrafish	N	Water	[29]
Quercetin	Renal function, dyslipidemia	Rat	N	Subcutaneous injection (Cd) and intraperitoneal injection (Quercetin)	[42]
Quercetin	Apoptosis, body weight, renal coefficient, oxidative stress, kidney tissue damage	Rat	N	Intraperitoneal injection	[43]
Quercetin	Apoptosis and oxidative stress in liver	Rat	N	Intraperitoneal injection, gavage	[44]
Quercetin	Apoptosis and oxidative stress in kidney	Rat	N	Intraperitoneal injection, oral	[45]
Quercetin	Effects on shoaling behavior, anxiety behavior, oxidative stress, neuroinflammation, neuron apoptosis	Zebrafish	N	Water	[46]
Vitamin E	Apoptosis, testes	Rat	N	Intraperitoneal injection	[47]
Vitamin E	Renal oxidative damage, apoptosis	Rat	N	Gavage	[48]
Modified and unmodified fingered citron peel pectin	Apoptosis, oxidative stress to kidney and liver	Mouse	N	Gavage	[49]
Red carrot methanolic extract or Vitamin E	Apoptosis, oxidative stress in testes, histopathological changes in testicular, prostatic, and semen vesicle glandular tissue	Rat	N	Gavage	[50]
Vitamin E	Oxidative stress in liver	Rat	N	Diet (Cd), intraperitoneal injection (Vitamin E)	[51]
Vitamin D	Nephrotoxicity	Rat	N	Gavage, drinking water	[52]
Vitamins C and E	Oxidative stress in kidney and liver	Rat	N	Drinking water, diet, and intraperitoneal injection	[53]
Selenium and Vitamin C	Body weight, accumulation of Cd in kidney and liver	Rat	N	Drinking water (Cd. Se, Vitamin C), intraperitoneal injection (Se and Vitamin C) and gavage (Cd)	[54]
Ajwain extract (*Trachyspermum ammi L.*)	Apoptosis and oxidative stress	PC12 cells	N	Water	[55]
Probiotic mixture (*Lactobacillus* (*L*.) *paracasei*, *L. plantarum*, *L. acidophilus*, *L. delbrueckii subsp. bulgaricus*, *bifidobacteria* (*B. longum, B. breve*, and *B. infantis*), and *Streptococcus thermophilus*)	Reduced Cd levels in infant stools, but not in breastmilk	Nursing mothers and infants	Y	Oral	[56]
*Lactobacillus plantarum* strains (CCFM8610, CCFM11, and CCFM309)	Cd excretion in bile and feces	Mouse	N	Drinking water (Cd), Oral (probiotics)	[57]
*Lactobacillus plantarum* strains (CCFM8610, CCFM11, and CCFM309)	Cd levels in blood	Human	Y	Oral	[57]
*Lactobacillus rhamnosus* GR-1	Immobilization of Cd and Pb, reducing translocation across intestinal epithelium	Caco-2 cell line (model of human intestinal epithelium)	N	Water	[58]
*Pediococcus pentosaceus* GS4	Cd excretion in feces; tissue accumulation	Mice	N	Gavage	[59]

Probiotics have shown promise in reducing Cd toxicity. Probiotic administration provide protection for those directly treated with protection extending to fetuses of exposed mothers [92, 93]. Specific strains of *Lactobacillus plantarum* have demonstrated the ability to reduce Cd levels in blood and increase Cd excretion in bile and feces in human clinical trials [94]. *Lactobacillus rhamnosus* GR-1 has also shown potential for immobilizing Cd and Pb, reducing their translocation across the intestinal epithelium in vitro using the human Caco-2 cell line [95].

Zinc and selenium are two divalent cations that have emerged as promising therapeutic agents against Cd toxicity. Zinc supplementation mitigates Cd toxicity through direct competition with Cd for binding sites, induction of metallothionein, and maintenance of redox homeostasis [96]. Selenium (Se), an essential nutrient for humans, is known to have neuroprotective effects in the brain from the incorporation of selenoproteins, an antioxidant [97, 98]. Selenium acts as an antagonist to Cd by enhancing antioxidant capacity, reducing inflammation, and modulating metal transport and steroidogenic gene expression [99]. Se pre-treatment alleviates Cd-induced bradycardia in zebrafish [46]. Selenium and vitamin C co-supplementation has also demonstrated protective effects against Cd toxicity, reducing body weight loss and Cd accumulation in rat kidney and liver [100]. Other vitamins have been shown to alleviate Cd-induced toxicity in the testes, kidneys and liver through reducing oxidative stress [101]. Interestingly, while zinc leads to Cd depletion and rescues Cd triggered toxicity in cholinergic neurons, selenium accumulation parallels Cd accumulation and fails to rescue Cd-induced toxicity in cholinergic neurons, suggesting that their neuroprotective effects are dependent on neuronal phenotype, and more broadly the organ [102].

Chelation therapy is commonly used to treat Cd toxicity. The primary intervention for cadmium toxicity involves chelation therapy using Edetate (EDTA), 2,3-dimercapto-l-propanesulfonic acid, Na salt (DMPS), or Meso-dimercaptosuccinic acid (DMSA). DMSA demonstrates superior cadmium removal compared to DMPS in animal models, while EDTA shows greater intracellular cadmium mobilization than DMSA in both in vitro and in vivo studies [91]. EDTA, the preferred clinical agent, achieves optimaltherapeutic outcomes when combined with antioxidants such as glutathione, vitamins E and C, methionine, mannitol, thiamine, and zinc, which provide nephroprotection [91]. In a recent human clinical trial, repeated EDTA infusions lead to short-term mobilization of urinary Cd but no decline levels over trial period, which contrast with its effects on lead, which exhibited reductions overtime [103]. While the mechanisms governing this metal dependent difference in EDTA mobilization remain elusive, the zebrafish is amenable to uncovering the mechanisms so that chelation therapies can be refined and new therapeutic targets can be identified.

Antioxidants and anti-inflammatory compounds have shown potential in mitigating Cd toxicity. Quercetin, an antioxidant and anti-inflammatory flavonoid, has been shown to reduce anxious behaviors associated with inflammation in Cd-exposed zebrafish [104] improve renal function and dyslipidemia in rats [105], protect against apoptosis and oxidative stress in rat liver and kidney, [106, 107] and alleviate Cd-induced cytotoxicity and apoptosis in PC12 cells [108]. The combination of *Tamarindus indica* and coenzyme Q10 (CoQ) has also demonstrated hepatorenal protection against Cd toxicity in rats, likely due to their synergistic antioxidant effects [90].

Gene therapy approaches, such as overexpression of the *atp6v0cb* gene, have shown promise in alleviating Cd-induced neurotoxicity in zebrafish larvae by restoring locomotor activity and reducing neuronal apoptosis [60]. The study found that Cd exposure downregulated genes in the V-ATPase family, which was associated with disturbance of the gut microbiota. Overexpression of atp6v0cb partially rescued the neurotoxic effects, suggesting the V-ATPase family plays a key role in cadmium toxicity mediated by the gut-brain axis [60]. Further research is needed to refine gene therapy strategies in terms of dose, timing, and potential off-target effects, and zebrafish models provide a valuable platform for high-throughput screening and optimization of these therapies.

While some therapies have shown success in humans, rats, and zebrafish, more refinement in terms of the concentration and timing of treatments are needed. Furthermore, while some systems or organs may benefit by the addition of therapeutics, additional testing is needed to evaluate off-target effects of each therapy. Zebrafish with high-throughput screening has the power to aid in the development of effective therapeutic strategies [109]. Our review demonstrates the value of zebrafish in advancing therapeutic development for various inflammatory diseases, even if an exact number of therapies is not specified. The rapid screening abilities of zebrafish make them well-suited for identifying and refining novel anti-inflammatory and neuroprotective treatments in the future.

## Conclusions

The findings from zebrafish studies provide valuable insights into the molecular and cellular mechanisms underlying cadmium-induced behavioral deficits, visual defects, cardiovascular dysfunction, and neurotoxicity, with implications for human health. At a molecular level, cadmium exposure disrupts crucial signaling pathways, particularly the Wnt/β-catenin pathway, alters calcium homeostasis, perturbs ionic channels such as Ca^2+^—ATPases, induces oxidative stress and inflammation leading to neuronal apoptosis, cell cycle arrest, and cardiovascular dysfunction [53, 54]. More recent proposed mechanisms of cadmium toxicity include gut microbiome dysbiosis [14, 58, 60]. The identification of reliable biomarkers, such as altered enzyme activities and gene expression changes, can aid in predicting individual susceptibility and monitoring therapeutic responses [28, 65, 67].

Translating findings from zebrafish studies to human applications is an essential aspect of enhancing mechanistic understanding and advancing therapeutic strategies. Epidemiological and animal studies link adverse reproductive effects with Cd exposure with some effects persisting across generations, but the human data is correlative, and the relevance of animal studies were limited because the route of exposure was water (not diet), exposure was acute, the concentrations used were are not environmentally relevant and behavior and cardiovascular function were not assessed as endpoints [30, 38, 80, 81, 89]. Moreover, many studies confuse inter- (F0-F1) with trans-generational effects (F0-F2), which precludes understanding of epigenetic exposures [30]. Sensory systems are the gateway for behavior. Thus, there is a need to examine Cd-induced sensory-behavioral and cardiovascular disorders across generations. While some therapies have shown success in zebrafish, rodents, and humans, further research is needed to refine these treatments in terms of dosage, timing, and potential off-target effects across multiple organ systems. Moreover, the accumulation of cadmium in zebrafish occurs in an organ-specific manner, with the gills, liver, brain, and gonads being particularly susceptible [20]. This highlights the importance of considering organ-specific toxicity when evaluating potential therapies. Importantly, cadmium exposure can result in neurotoxicity, genotoxicity, and epigenetic alterations even without causing significant accumulation within the organism [110]. This underscores the need for high-throughput screening of body and tissue burden analysis to better understand the toxicological profile of Cd and link phenotypic endpoints to bioaccumulation patterns. Synergistic effects of climate change (e.g., increasing temperature, more frequent and extreme droughts), soil and water acidification, changes in land use, and other anthropogenic forces will impact the toxicity and distribution of Cd and other legacy and emerging contaminants [111, 112]. These anthropogenic effects necessitate the study of cadmium toxicity in more ecologically relevant settings with environmentally relevant concentrations and exposure routes.

## Key References

- Genchi G, Sinicropi MS, Lauria G, Carocci A, Catalano A. The effects of cadmium toxicity. *Int J Environ Res Public Health*. 2020;17(11):3782. 10.3390/ijerph17113782.
  - ○ Reviews Cd properties, molecular and epigenetic mechanisms of cadmium toxicity and potential therapeutics and phytoremediation strategies.
- Xu Y, Yu Y, Zhou Q, et al. Disturbance of gut microbiota aggravates cadmium-induced neurotoxicity in zebrafish larvae through V-ATPase. *Sci Total Environ*. 2023;891:164074. 10.1016/j.scitotenv.2023.164074.
  - ○ Identifies a novel connection between gut microbiota perturbations and Cd-induced neurotoxicity in zebrafish.
- Zhang Y, Feng J, Gao Y, Liu X, Qu L, Zhu L. Physiologically based toxicokinetic and toxicodynamic (PBTK-TD) modelling of Cd and Pb exposure in adult zebrafish *Danio rerio*: Accumulation and toxicity. *Environ Pollut*. 2019;249:959–968. 10.1016/j.envpol.2019.03.115.
  - ○ Documents bioaccumulation of Cd in various organs and develops a toxicodynamic model to predict accumulation patterns in zebrafish.
- Pierron F, Daramy F, Heroin D, et al. Sex-specific DNA methylation and transcription of zbtb38 and effects of gene–environment interactions on its natural antisense transcript in zebrafish. *Epigenetics*. 2023;18(1):2260963. 10.1080/15592294.2023.2260963.
  - ○ Identified crucial genes and altered methylation patterns related to transgenerational effect of Cd exposure in zebrafish. First study to demonstrate transgenerational effects of Cd toxicity in zebrafish and identify potential epigenetic mechanisms.
- Xu Y, Liu J, Tian Y, et al. Wnt/β-catenin signaling pathway Is strongly implicated in cadmium-induced developmental neurotoxicity and neuroinflammation: Clues from zebrafish neurobehavior and *in vivo* neuroimaging. *Int J Mol Sci*. 2022;23(19). 10.3390/ijms231911434.
  - ○ Demonstrates that modulating Wnt/β-catenin pathway can mitigate some of the harmful effects of Cd-induce neurotoxicity.

## Supplementary Information

Below is the link to the electronic supplementary material.

Supplementary file1 (DOCX 20 KB)

## Data Availability

No datasets were generated or analysed during the current study.
