# Supplementary material for: Swimming Into View: Zebrafish Uncover Targets, Mechanisms, and Therapies for Cadmium Toxicity
Source: Curr Environ Health Rep. 2025 Apr 22;12(1):20. doi: 10.1007/s40572-025-00471-0 (PMC12011950; doi:10.1007/s40572-025-00471-0)
Supplement: Supplementary file 1 — Supplementary file1 (DOCX 20 KB) [file 40572_2025_471_MOESM1_ESM.docx]

**Supplementary Materials**

The following references are related to material presented in Tables 1-3.

**References**

1. Gonzalez P, Baudrimont M, Boudou A, Bourdineaud JP. Comparative effects of direct cadmium contamination on gene expression in gills, liver, skeletal muscles and brain of the zebrafish (*Danio rerio*). *Biometals*. 2006;19(3):225-235. doi:10.1007/s10534-005-5670-x

2. Favorito R, Chiarelli G, Grimaldi MC, De Bonis S, Lancieri M, Ferrandino I. Bioaccumulation of cadmium and its cytotoxic effect on zebrafish brain. *Chemistry and Ecology*. 2011;27(sup2):39-46. doi:10.1080/02757540.2011.625937

3. Petrova E, Pashkunova-Martic I, Schaier M, et al. Effects of subacute cadmium exposure and subsequent deferiprone treatment on cadmium accumulation and on the homeostasis of essential elements in the mouse brain. *Journal of Trace Elements in Medicine and Biology*. 2022;74:127062. doi:10.1016/j.jtemb.2022.127062

4. Choudhuri S, Li Liu W, Berman NEJ, Klaassen CD. Cadmium accumulation and metallothionein expression in brain of mice at different stages of development. *Toxicology Letters*. 1996;84(3):127-133. doi:10.1016/0378-4274(95)03444-7

5. Lech T, Sadlik JK. Cadmium concentration in human autopsy tissues. *Biol Trace Elem Res*. 2017;179(2):172-177. doi:10.1007/s12011-017-0959-5

6. Abd-Elhakim YM, El Bohi KM, El Sharkawy NI, Ghali MA, Haseeb S. The impacts of individual and combined exposure to cadmium and lead on intraocular pressure, electroretinography, and residual changes in the rabbit eyes. *Environ Sci Pollut Res*. 2019;26(32):33321-33328. doi:10.1007/s11356-019-06446-7

7. Erie JC, Butz JA, Good JA, Erie EA, Burritt MF, Cameron JD. Heavy metal concentrations in human eyes. *American Journal of Ophthalmology*. 2005;139(5):888-893. doi:10.1016/j.ajo.2004.12.007

8. Ashraf W. Accumulation of heavy metals in kidney and heart tissues of *Epinephelus microdon*  fish from the Arabian Gulf. *Environmental monitoring and assessment*. 2005;101:311-316.

9. Soares SS, Martins H, Gutiérrez-Merino C, Aureliano M. Vanadium and cadmium *in vivo* effects in teleost cardiac muscle: Metal accumulation and oxidative stress markers. *Comparative Biochemistry and Physiology Part C: Toxicology & Pharmacology*. 2008;147(2):168-178. doi:10.1016/j.cbpc.2007.09.003

10. Tai YT, Chou SH, Cheng CY, et al. The preferential accumulation of cadmium ions among various tissues in mice. *Toxicology Reports*. 2022;9:111-119. doi:10.1016/j.toxrep.2022.01.002

11. Banni M, Chouchene L, Said K, Kerkeni A, Messaoudi I. Mechanisms underlying the protective effect of zinc and selenium against cadmium-induced oxidative stress in zebrafish Danio rerio. *Biometals*. 2011;24(6):981-992. doi:10.1007/s10534-011-9456-z

12. Zhang Y, Feng J, Gao Y, Liu X, Qu L, Zhu L. Physiologically based toxicokinetic and toxicodynamic (PBTK-TD) modelling of Cd and Pb exposure in adult zebrafish *Danio rerio*: Accumulation and toxicity. *Environmental Pollution*. 2019;249:959-968. doi:10.1016/j.envpol.2019.03.115

13. Jancová A, Massányi P, Nad P, et al. Accumulation of heavy metals in selected organs of yellow-necked mouse (*Apodemus Flavicollis*). *Ekologia(Bratislava)/Ecology(Bratislava)*. 2006;25(1):19-26.

14. Egger AE, Grabmann G, Gollmann-Tepeköylü C, et al. Chemical imaging and assessment of cadmium distribution in the human body†. *Metallomics*. 2019;11(12):2010-2019. doi:10.1039/c9mt00178f

15. Kusakabe T, Nakajima K, Suzuki K, et al. The changes of heavy metal and metallothionein distribution in testis induced by cadmium exposure. *Biometals*. 2008;21(1):71-81. doi:10.1007/s10534-007-9094-7

16. Zhou J, Zeng L, Zhang Y, et al. Cadmium exposure induces pyroptosis in testicular tissue by increasing oxidative stress and activating the AIM2 inflammasome pathway. *Science of The Total Environment*. 2022;847:157500. doi:10.1016/j.scitotenv.2022.157500

17. Charkiewicz AE, Omeljaniuk WJ, Nowak K, Garley M, Nikliński J. Cadmium Toxicity and Health Effects—A Brief Summary. *Molecules*. 2023;28(18):6620. doi:10.3390/molecules28186620

18. Yang H, Zhu Z, Xie Y, et al. Comparison of the combined toxicity of polystyrene microplastics and different concentrations of cadmium in zebrafish. *Aquatic Toxicology*. 2022;250:106259. doi:10.1016/j.aquatox.2022.106259

19. Madesh S, Sudhakaran G, Ramamurthy K, et al. Cadmium and ketoprofen accumulation influences aquatic ecosystem demonstrated using *in-vivo* zebrafish model. *Drug and Chemical Toxicology*. Published online June 23, 2024:1-16. doi:10.1080/01480545.2024.2364240

20. McKenna IM, Waalkes MP, Chen LC, Gordon T. Comparison of inflammatory lung responses in Wistar rats and C57 and DBA mice following acute exposure to cadmium oxide fumes. *Toxicology and Applied Pharmacology*. 1997;146(2):196-206. doi:10.1006/taap.1997.8241

21. Matz CJ, Treble RG, Krone PH. Accumulation and elimination of cadmium in larval stage zebrafish following acute exposure. *Ecotoxicology and Environmental Safety*. 2007;66(1):44-48. doi:10.1016/j.ecoenv.2005.11.001

22. Burnison BK, Meinelt T, Playle R, Pietrock M, Wienke A, Steinberg CE. Cadmium accumulation in zebrafish (*Danio rerio*) eggs is modulated by dissolved organic matter (DOM). *Aquatic toxicology*. 2006;79(2):185-191.

23. Renieri EA, Sfakianakis DG, Alegakis AA, et al. Nonlinear responses to waterborne cadmium exposure in zebrafish. An in vivo study. *Environmental research*. 2017;157:173-181.

24. Xu Y, Yu Y, Zhou Q, et al. Disturbance of gut microbiota aggravates cadmium-induced neurotoxicity in zebrafish larvae through V-ATPase. *Science of The Total Environment*. 2023;891:164074. doi:10.1016/j.scitotenv.2023.164074

25. Yang J, Li J, Zhang X, et al. Effects of ecologically relevant concentrations of cadmium on the microbiota, short-chain fatty acids, and FFAR2 expression in zebrafish. *Metabolites*. 2023;13(5). doi:10.3390/metabo13050657

26. Xia Y, Zhu J, Xu Y, Zhang H, Zou F, Meng X. Effects of ecologically relevant concentrations of cadmium on locomotor activity and microbiota in zebrafish. *Chemosphere*. 2020;257:127220. doi:10.1016/j.chemosphere.2020.127220

27. Xu Y, Liu J, Tian Y, et al. Wnt/β-catenin signaling pathway Is strongly implicated in cadmium-induced developmental neurotoxicity and neuroinflammation: Clues from zebrafish neurobehavior and *in vivo* neuroimaging. *International Journal of Molecular Sciences*. 2022;23(19). doi:10.3390/ijms231911434

28. Huang Y, Dai Y, Li M, et al. Exposure to cadmium induces neuroinflammation and impairs ciliogenesis in hESC-derived 3D cerebral organoids. *Science of The Total Environment*. 2021;797:149043. doi:10.1016/j.scitotenv.2021.149043

29. Li Y, Wang R, Li Y, Sun G, Mo H. Protective effects of tree peony seed protein hydrolysate on Cd-induced oxidative damage, inflammation and apoptosis in zebrafish embryos. *Fish & Shellfish Immunology*. 2022;126:292-302. doi:10.1016/j.fsi.2022.05.033

30. Patel UN, Patel UD, Khadayata AV, Vaja RK, Patel HB, Modi CM. Assessment of neurotoxicity following single and co-exposure of cadmium and mercury in adult zebrafish: Behavior alterations, oxidative Stress, gene expression, and histological impairment in brain. *Water, Air, & Soil Pollution*. 2021;232(8):340. doi:10.1007/s11270-021-05274-1

31. Bian X, Gao Y. DNA methylation and gene expression alterations in zebrafish embryos exposed to cadmium. *Environ Sci Pollut Res*. 2021;28(23):30101-30110. doi:10.1007/s11356-021-12691-6

32. Guo SN, Zheng JL, Yuan SS, Zhu QL. Effects of heat and cadmium exposure on stress-related responses in the liver of female zebrafish: Heat increases cadmium toxicity. *Sci Total Environ*. 2018;618:1363-1370. doi:10.1016/j.scitotenv.2017.09.264

33. Wang CC, Si LF, Guo SN, Zheng JL. Negative effects of acute cadmium on stress defense, immunity, and metal homeostasis in liver of zebrafish: The protective role of environmental zinc dpre-exposure. *Chemosphere*. 2019;222:91-97. doi:10.1016/j.chemosphere.2019.01.111

34. Yang Y, Ye X, He B, Liu J. Cadmium potentiates toxicity of cypermethrin in zebrafish. *Environmental Toxicology and Chemistry*. 2016;35(2):435-445. doi:10.1002/etc.3200

35. Tarasco M, Cardeira J, Viegas MN, et al. Anti-osteogenic activity of cadmium in zebrafish. *Fishes*. 2019;4(1):11. doi:10.3390/fishes4010011

36. Chen YY, Zhu JY, Chan KM. Effects of cadmium on cell proliferation, apoptosis, and proto-oncogene expression in zebrafish liver cells. *Aquatic Toxicology*. 2014;157:196-206. doi:10.1016/j.aquatox.2014.10.018

37. Hu W, Zhu QL, Zheng JL, Wen ZY. Cadmium induced oxidative stress, endoplasmic reticulum (ER) stress and apoptosis with compensative responses towards the up-regulation of ribosome, protein processing in the ER, and protein export pathways in the liver of zebrafish. *Aquatic Toxicology*. 2022;242:106023. doi:10.1016/j.aquatox.2021.106023

38. Abdelnaby A, Abdel-Aleem N, Mansour A, et al. The combination of *tamarindus indica* and coenzyme Q10 can be a potential therapy preference to attenuate cadmium-induced hepatorenal injury. *Frontiers in Pharmacology*. 2022;13. https://www.frontiersin.org/journals/pharmacology/articles/10.3389/fphar.2022.954030

39. Devarapogu R, Asupatri UR. Effects of zinc supplementation in mitigating the harmful effects of chronic cadmium exposure in a zebrafish model. *Environmental Toxicology and Pharmacology*. 2023;100. doi:10.1016/j.etap.2023.104158

40. Branca JJV, Morucci G, Maresca M, et al. Selenium and zinc: Two key players against cadmium-induced neuronal toxicity. *Toxicology in Vitro*. 2018;48:159-169. doi:10.1016/j.tiv.2018.01.007

41. Heuer RM, Falagan-Lotsch P, Okutsu J, et al. Therapeutic efficacy of selenium pre-treatment in mitigating cadmium-induced cardiotoxicity in zebrafish (*Danio rerio*). Published online July 3, 2024. doi:10.21203/rs.3.rs-4583781/v1

42. Hatipoğlu D, Özsan M, Kısadere İ, Dönmez N. Quaercetin improves renal functional disorder and dyslipidemia caused by acute cadmium exposure. *MJAVL*. 2023;13(1):50-58. doi:10.53518/mjavl.1196166

43. Ding L, Wang K, Zhu H, Liu Z, Wang J. Protective effect of quercetin on cadmium-induced kidney apoptosis in rats based on PERK signaling pathway. *Journal of Trace Elements in Medicine and Biology*. 2024;82:127355. doi:10.1016/j.jtemb.2023.127355

44. Wang J, Ding L, Wang K, et al. Role of endoplasmic reticulum stress in cadmium-induced hepatocyte apoptosis and the protective effect of quercetin. *Ecotoxicology and environmental safety*. 2022;241:113772-113772. doi:10.1016/j.ecoenv.2022.113772

45. Huang R, Ding L, Ye Y, et al. Protective effect of quercetin on cadmium-induced renal apoptosis through cyt-c/caspase-9/caspase-3 signaling pathway. *Frontiers in pharmacology*. 2022;13:990993-990993. doi:10.3389/fphar.2022.990993

46. Zhang J liang, Liu M, Cui W, Yang L, Zhang C nuan. Quercetin affects shoaling and anxiety behaviors in zebrafish: Involvement of neuroinflammation and neuron apoptosis. *Fish & Shellfish Immunology*. 2020;105:359-368. doi:10.1016/j.fsi.2020.06.058

47. Amanpour P, Khodarahmi P, Salehipour M. Protective effects of vitamin E on cadmium-induced apoptosis in rat testes. *Naunyn-Schmiedeberg’s Arch Pharmacol*. 2020;393(3):349-358. doi:10.1007/s00210-019-01736-w

48. Fang J, Xie S, Chen Z, et al. Protective effect of vitamin E on cadmium-induced renal oxidative damage and apoptosis in rats. *Biol Trace Elem Res*. 2021;199(12):4675-4687. doi:10.1007/s12011-021-02606-4

49. Ying X, Pan Y, Lan J, Fang Y, Ding YY, Gu Z. Preparation of pectin from fingered citron peel and its protective effect on cadmium-induced liver and kidney damage in mice. *Food Bioscience*. 2023;56:103359. doi:10.1016/j.fbio.2023.103359

50. Abdel-Wahab A, Hassanin KMA, Mahmoud AA, et al. Physiological roles of red carrot methanolic extract and vitamin E to abrogate cadmium-induced oxidative challenge and apoptosis in rat testes: involvement of the Bax/Bcl-2 ratio. *Antioxidants*. 2021;10(11):1653. doi:10.3390/antiox10111653

51. Bahri S, Kaddour H, Karoui D, Bouraoui S, Amri M, Mokni M. Protective role of vitamin E against cadmium induced oxidative stress into the rat liver. *Tunisie Medicale*. 2019;97(1):100-105.

52. Abdelghany AH, Refaat B, Hassan AA, Abd  ElAziz M. The protective effects of Vitamin D against Cadmium-induced nephrotoxicity. *Journal of Trace Elements and Minerals (Online)*. 2023;6:100097. doi:10.1016/j.jtemin.2023.100097

53. Poli V, Aparna Y, Madduru R, Motireddy SR. Protective effect of vitamin C and E on enzymatic and antioxidant system in liver and kidney toxicity of Cadmium in rats. *Applied Food Research*. 2022;2(1):100098. doi:10.1016/j.afres.2022.100098

54. Zamani MM, Mortazavi SH, Monajjemzadeh M, Piranfar V, Aalidaeijavadi Z, Bakhtiarian A. Protective effect of combined long time administration of selenium and vitamin C on liver and kidney toxicity of cadmium in rats. *Iranian journal of pathology*. 2021;16(2):174-180. doi:10.30699/IJP.2020.135777.2489

55. Banik S, Rahman MdM, Sikder MdT, Saito T, Kurasaki M. Protective effects of ajwain (*Trachyspermum ammi L.*) extract against cadmium-induced cytotoxicity and apoptosis in PC12 cells. *Journal of herbal medicine*. 2021;26:100423. doi:10.1016/j.hermed.2021.100423

56. Astolfi ML, Protano C, Schiavi E, et al. A prophylactic multi-strain probiotic treatment to reduce the absorption of toxic elements: In-vitro study and biomonitoring of breast milk and infant stools. *Environment international*. 2019;130:104818. doi:10.1016/j.envint.2019.05.012

57. Zhai Q, Liu Y, Wang C, et al. Increased cadmium excretion due to oral administration of *Lactobacillus plantarum* strains by regulating enterohepatic circulation in mice. *J Agric Food Chem*. 2019;67(14):3956-3965. doi:10.1021/acs.jafc.9b01004

58. Daisley BA, Monachese M, Trinder M, et al. Immobilization of cadmium and lead by *Lactobacillus rhamnosus* GR-1 mitigates apical-to-basolateral heavy metal translocation in a Caco-2 model of the intestinal epithelium. *Gut microbes*. 2019;10(3):321-333. doi:10.1080/19490976.2018.1526581

59. Dubey V, Mishra AK, Ghosh AR, Mandal BK. Probiotic Pediococcus pentosaceus GS4 shields brush border membrane and alleviates liver toxicity imposed by chronic cadmium exposure in Swiss albino mice. *Journal of applied microbiology*. 2019;126(4):1233-1244. doi:10.1111/jam.14195
